# Experimental and Theoretical Studies on the Adsorption of Bromocresol Green from Aqueous Solution Using Cucumber Straw Biochar

**DOI:** 10.3390/molecules29194517

**Published:** 2024-09-24

**Authors:** Chenxi Zhang, Lingbin Meng, Zhihao Fang, Youxin Xu, Yue Zhou, Hongsen Guo, Jinyu Wang, Xiaotian Zhao, Shuyan Zang, Hailin Shen

**Affiliations:** 1 Shandong Provincial University Laboratory for Protected Horticulture, Weifang University of Science and Technology, Weifang 262700, China; morningsunzhang@outlook.com (C.Z.); mlb8124@126.com (L.M.); xuyouxin@wfust.edu.cn (Y.X.); 17854072321@163.com (Y.Z.); g2037@outlook.com (H.G.); jinyu678678@outlook.com (J.W.); zhaoxiaotian0520@outlook.com (X.Z.); 2 College of Science, Shenyang University of Chemical Technology, Shenyang 110142, China; zangshuyan@126.com; 3 School of Chemical Engineering and Materials, Changzhou Institute of Technology, Changzhou 213032, China

**Keywords:** cucumber straw biochar, bromocresol green, adsorption mechanism, density functional theory

## Abstract

Biochar prepared from crop straw is an economical method for adsorbing bromocresol green (BCG) from textile industrial wastewater. However, there is limited research on the adsorption mechanism of biochar for the removal of BCG. This study utilized cucumber straw as raw material to prepare biochar with good adsorption potential and characterized its physicochemical properties. Through adsorption experiments, the effects of solution pH, biochar dosage, and initial dye concentration on adsorption performance were examined. The adsorption mechanism of cucumber straw biochar (CBC) for BCG was elucidated at the molecular level using adsorption kinetics, adsorption isotherm models, and density functional theory (DFT) calculations. Results show that the specific surface area of the CBC is 101.58 m^2^/g, and it has a high degree of carbonization, similar to the structure of graphite crystals. The presence of aromatic rings, –OH groups, and –COOH groups in CBC provides abundant adsorption sites for BCG. The adsorption process of CBC for BCG is influenced by both physical and chemical adsorption, and can be described by the Langmuir isotherm model, indicating a monolayer adsorption process. The theoretical maximum monolayer adsorption capacity (q_m_) of BCG at 298 K was calculated to be 99.18 mg/g. DFT calculations reveal interactions between BCG and CBC involving electrostatic interactions, van der Waals forces, halogen–π interactions, π–π interactions, and hydrogen bonds. Additionally, the interaction of hydrogen bonds between BCG and the –COOH group of biochar is stronger than that between BCG and the –OH group. These findings provide valuable insights into the preparation and application of efficient organic dye adsorbents.

## 1. Introduction

Bromocresol green (BCG) is a dye belonging to the triphenylmethane family. Besides its applications in medicine, microbiology, and environmental testing, it is primarily used in the textile industry for dyeing wool, silk, nylon, and polyamide [1,2,3]. BCG is commonly released in textile industrial wastewater. Due to its complex aromatic molecular structure and difficulty in biological degradation, it causes severe environmental pollution [4]. The presence of BCG dye in water can reduce light penetration, thereby decreasing photosynthesis and causing harm to aquatic organisms [5]. Furthermore, it can impact aquatic life at low concentrations and has been proven to be a carcinogen [6,7]. Therefore, employing suitable and effective methods for treating textile industrial wastewater is of great importance.

Although various techniques, such as chemical oxidation [8,9], electrocatalysis [10], and photocatalysis [11,12] have been successfully applied to treat BCG-containing industrial wastewater, adsorption has proven to be a mature and cost-effective method due to its low cost, high efficiency, and ease of design [13,14]. To date, various adsorbents have been prepared and used to remove BCG from wastewater, such as modified zeolites [15], chitosan-based polymethyl methacrylate [5], magnetic nanoparticles [16], and bimetallic organic frameworks [17]. However, their complex preparation processes and high costs limit their practical application. Recently, adsorbents derived from natural, industrial, and agricultural waste have gained popularity [18]. Among them, biochar prepared from crop straw has attracted significant attention due to its low cost and high pollutant removal efficiency. Phuong et al. prepared biochar from rice straw and rice husks to investigate its adsorption capacity for BCG and explore its potential adsorption mechanisms [19]. Kaya and Uzun investigated the potential application of pine cone-derived activated biochar for removing BCG dye from water [20]. However, these studies often use a combination of adsorption models and spectroscopic analysis to investigate adsorbent performance and interaction mechanisms. For instance, adsorption rate constants are calculated using pseudo-first order and pseudo-second order kinetic models; maximum adsorption capacities are determined using Langmuir and Freundlich models; and Fourier transform infrared spectroscopy (FTIR) is used to infer bonding mechanisms between active sites and pollutants during the adsorption process. Nevertheless, these analytical methods are difficult to reveal the interaction mechanisms between adsorbents and pollutants from an atomic perspective.

In recent years, density functional theory (DFT) calculations have been widely used to describe the structural, electronic, and energy characteristics of interactions between adsorbents and adsorbates due to their high accuracy, short computation time, and low cost [21,22,23]. Cheng et al. prepared N-doped porous graphitized biochar from alfalfa and used DFT calculations to further understand the crucial role of the N configuration, analyzing the interactions between methylene blue (MB) and methyl orange with the biochar at the molecular and electronic levels [24]. Zhou et al. elucidated the adsorption mechanism of MB on citrus residue biochar at the molecular level through adsorption experiments, adsorption models, and DFT calculations [25]. The study found that the adsorption mechanism depends on electrostatic forces, hydrogen bonds, π–π interactions, and van der Waals forces formed between –COOH, –OH, and –NH–CO– groups in the biochar structure and the amino groups in MB. Therefore, establishing structural models of biochar through DFT calculations can accurately explain the interaction mechanisms during the adsorption process.

Cucumbers are one of the top ten most popular vegetable crops globally due to their high yield, good economic benefits, convenience, and rich nutrition. Since the 1970s, China has led the world in cucumber cultivation area and production, exceeding 70% of the global total [26]. However, the comprehensive utilization of cucumber straw is minimal, and cucumber straw waste is indiscriminately discarded, leading to wasted resources and significant agricultural pollution [27]. Cucumber straw mainly consists of cellulose, hemicellulose, and lignin, with a total content of C, H, and O exceeding 70%, indicating its potential for conversion into high-quality biochar with a high carbonization rate [28]. Nevertheless, research on the preparation of cucumber straw biochar and its application in dye wastewater treatment is limited.

In view of this, the aim of this study is to investigate the feasibility of using cucumber straw biochar (CBC) to adsorb BCG and to explore its potential adsorption mechanisms. Various techniques, including scanning electron microscopy (SEM), X-ray diffraction analysis (XRD), X-ray photoelectron spectroscopy (XPS), and FTIR, were used to characterize the structure and chemical properties of the biochar. The effects of the solution pH, biochar dosage, and initial dye concentration on adsorption performance were examined. Based on adsorption kinetics, adsorption isotherms, and DFT study results, the adsorption mechanisms of BCG by CBC were explored. This research provides theoretical and technical references for the resource utilization of vegetable waste in environmental protection.

## 2. Results and Discussion

### 2.1. Structural Characterization of Biochar

#### 2.1.1. Pore Structure Analysis

**Figure 1:**
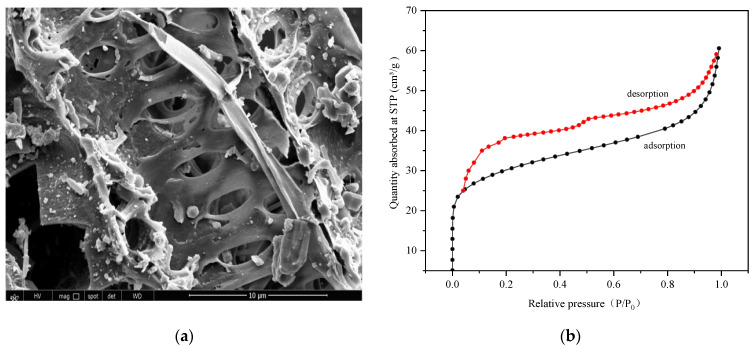
(**a**) SEM image, and (**b**) the N_2_ adsorption–desorption isotherms of CBC.

The microscopic morphological structure of CBC is shown in Figure 1a. Its surface is wrinkled, with a stable pore structure and noticeable porosity. There are some elliptical large pores with a size of about 1.5–3.5 µm, and many impurity particles are observed. These may include mineral components such as Ca, Si, and Mg from straw, which exist in the form of carbonates, phosphates, and oxides in biochar under high temperature [29,30]. The porous structure not only increases the specific surface area of the biochar, but also provides more adsorption sites and improves pore connectivity. As shown in Figure 1b, the adsorption rapidly approaches a linear increase in the low-pressure region (relative pressure less than 0.1). As the relative pressure continues to rise, adsorption still increases, but at a slower rate within a certain range. When P/P_0_ increases to a certain extent, capillary condensation occurs, accompanied by desorption hysteresis. According to IUPAC classification, CBC exhibits a typical IV N_2_ adsorption–desorption isotherm with an H4 hysteresis loop, indicating significant mesoporous structures [31].

The surface areas and the porosity structure were measured using the Brunauer–Emmett–Teller (BET) method, and the results are shown in Table 1. The specific surface area of the CBC is 101.58 m^2^/g, which is lower than that of the corn straw biochar (329.0 m^2^/g) prepared at same temperature [32]. However, CBC has a higher pore volume (0.0942 cm^3^/g) and smaller average pore size (1.85 nm), which could offer more active sites [33]. Table 1 also lists the elemental content of CBC, which contains 71.23% C and 3.12% H. Its H/C ratio is 0.044, which is less than 0.07, indicating higher biochemical stability and a high degree of aromatization [34].

#### 2.1.2. Powder X-ray Diffraction Studies

**Figure 2:**
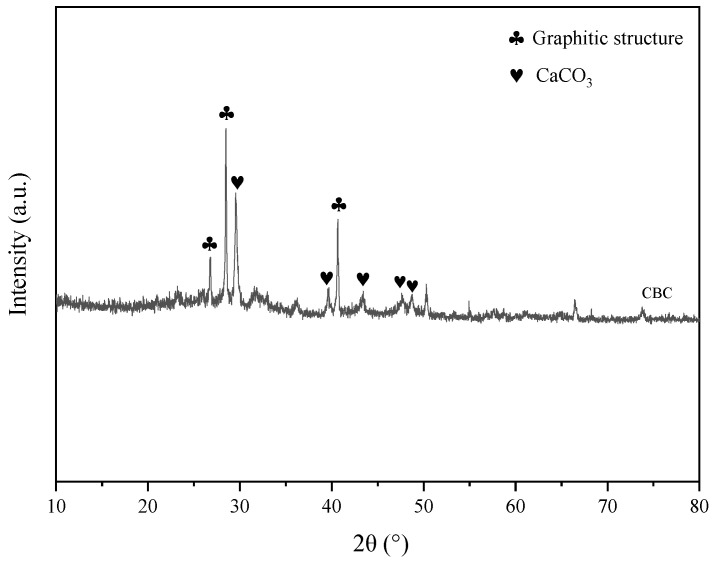
XRD patterns of CBC.

The crystal phase structures of CBC were characterized using XRD analyses, as displayed in Figure 2. Two peaks at 2θ = 26.62° and 28.47° correspond to the (002) plane of turbostratic graphite, indicating the presence of graphite crystal particles in the activated biochar. Additionally, another diffraction peak around 2θ = 41.20° corresponds to the graphite (100) plane, further confirming the graphite structure. The enhanced graphitic structure supports π-π interactions for BCG adsorption, suggesting a high degree of carbonization in the biochar similar to graphite crystal structures [35]. Peaks at 2θ = 29.54°, 39.58°, 43.42°, 47.60°, and 48.65° correspond to the symmetric calcite structure of CaCO_3_ (JCPDS 47-1743), which may be derived from the inherent components of cucumber straw [36].

#### 2.1.3. X-ray Photoelectron Spectroscopy and Fourier Transform Infrared Spectroscopy Analysis

**Figure 3:**
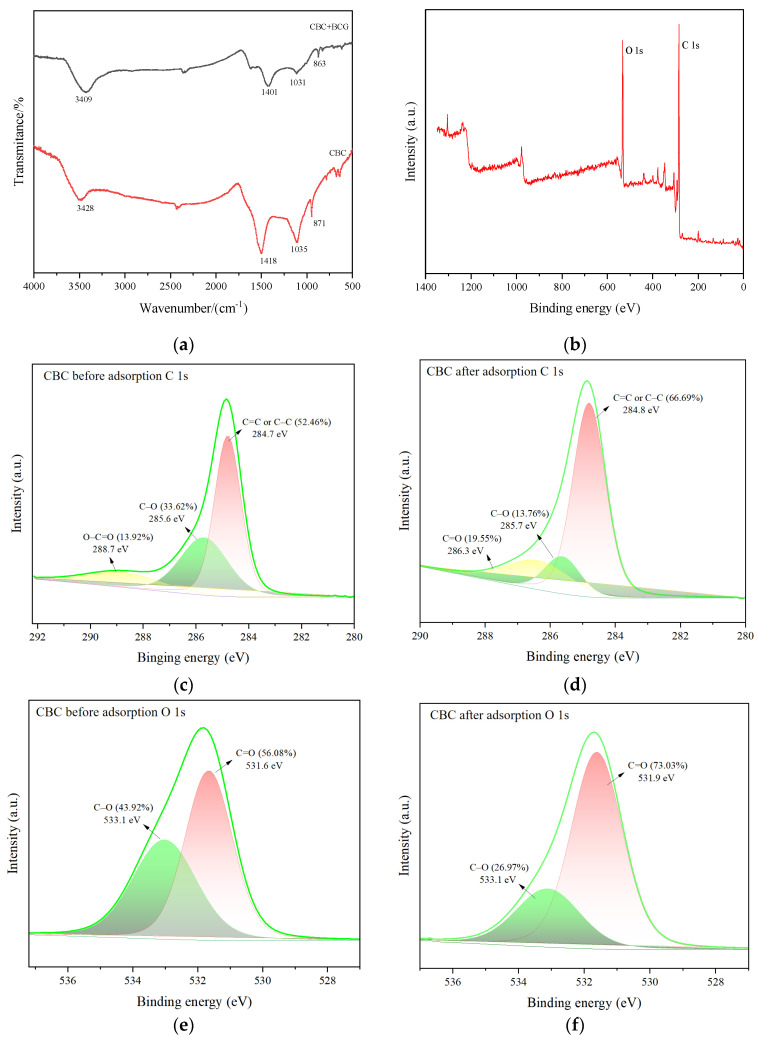
(**a**) The FT-IR spectra of CBC before and after the adsorption of BCG, (**b**) XPS spectra of CBC, (**c**) XPS core spectrum C 1s before adsorption, (**d**) XPS core spectrum C 1s after adsorption, (**e**) XPS core spectrum O 1s before adsorption, and (**f**) XPS core spectrum O 1s after adsorption.

To better understand the surface chemical structure of CBC, XPS analysis was conducted. Figure 3a shows the full scan XPS of CBC, revealing significant peaks for C 1s and O 1s, indicating the presence of C and O elements. Figure 3b displays the C 1s spectrum of CBC, which has three characteristic peaks at 284.7 eV (C=C or C–C, 52.46%), 285.6 eV (C–O, 33.62%), and 288.7 eV (O–C=O, 13.92%) [25]. The O 1s spectrum of CBC is shown in Figure 3c, where two peaks can be observed at 531.6 and 533.1 eV, belonging to C=O (56.08%) and C–O (43.92%), respectively. These results indicate that CBC contains oxygen-containing functional groups and a graphitic structure, which is consistent with the previous XRD findings.

FTIR was used to further analyze the oxygen-containing functional groups in the prepared biochar. As shown in Figure 3d, the CBC infrared spectrum was analyzed in the wavenumber range of 500–4000 cm^−1^. It was found that CBC observed an absorption peak near 3428 cm^−1^, which was mainly caused by the vibration of the –OH of phenols or alcohols, indicating the presence of phenolic or alcohol hydroxyl groups in CBC [37]. The peak around 1418 cm^−1^ is due to the symmetric stretching vibrations of C=O in carboxyl groups [38]. The peak near 1035 cm^−1^ results from the stretching vibrations of C–O in –OH and –COOH groups, while the peak at 871 cm^−1^ corresponds to the bending vibrations of C–H in aromatic rings [39]. These findings suggest the presence of aromatic rings, –OH groups, and –COOH groups in the biochar, providing abundant adsorption sites for BCG.

### 2.2. Adsorption Characteristics of CBC for BCG

#### 2.2.1. Effect of Initial Solution pH

**Figure 4:**
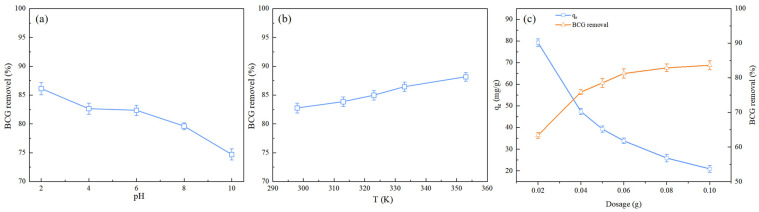
Effects of (**a**) pH, (**b**) temperature, and (**c**) dosage on the removal of BCG.

The pH value can affect the surface charge of the adsorbent and its chemical form in water, influencing the interactions between the adsorbent and dye molecules. Therefore, it is necessary to study the impact of pH on adsorption [40]. The removal efficiency of BCG by CBC varies with pH, as shown in Figure 4a. As the pH value increases, the removal efficiency decreases from 86.1% (pH = 2) to 74.6% (pH = 10). This trend is consistent with previous studies using cone-derived activated biochar and biochars produced from rice residues [19,20]. The change in removal efficiency can be explained by the pKa value of BCG (pKa = 4.7). Anionic BCG dyes exist in the form of negatively charged ions in aqueous solutions. At a low pH, the increase in H^+^ leads to the protonation of the CBC surface, enhancing the electrostatic interactions between the BCG and CBC [41]. At a higher pH, the surface of CBC carries strong negative charges, which create electrostatic repulsion with the negatively charged BCG molecules, reducing the amount of BCG adsorbed onto the CBC [42].

#### 2.2.2. Effect of Temperature

The influence of temperature on the adsorption process is complex, depending on the specific conditions and adsorbent materials involved [20]. Therefore, it is crucial to carefully study and optimize temperature conditions to achieve ideal adsorption performance. The effect of temperature on the removal of BCG by CBC was investigated in the range of 298–353 K, and the results are shown in Figure 4b. The results indicate that the removal efficiency of BCG increases with rising temperature. At room temperature (298 K), the removal efficiency is 82.7%, while at 353 K, it reaches 88.2%. This may be because, with increasing temperature, the kinetic energy of BCG molecules also increases, making them more likely to collide with and be adsorbed onto the CBC surface [43].

#### 2.2.3. Effect of Biochar Dosage

The dosage of the adsorbent is a key factor in evaluating the quality of an adsorbent, as it directly determines the economic benefits of the adsorbent [17]. To determine the effect of biochar dosage on the removal rate of BCG, the adsorption efficiency and capacity of BCG were studied at a BCG concentration of 50 mg/L, with varying amounts of biochar. The results are shown in Figure 4c. It can be observed that within the range of 0.01 to 0.1 g, the BCG adsorption efficiency gradually increased from 63.3% to 83.6% with the increase in biochar dosage. This is because an increase in CBC dosage leads to a higher number of available active adsorption sites, thereby enhancing the adsorption efficiency [44]. However, as the dosage of CBC increases, the adsorption capacity gradually decreases from 79.2 mg/g to 20.9 mg/g. This is due to the over-saturation of adsorption sites on the CBC surface, which results in a decline in unit adsorption capacity [45]. When the dosage exceeds 0.08 g, the adsorption efficiency tends to stabilize. Therefore, to achieve optimal adsorption efficiency, the best dosage of the adsorbent is determined to be 0.08 g.

### 2.3. Adsorption Model Analysis

#### 2.3.1. Adsorption Kinetics

**Figure 5:**
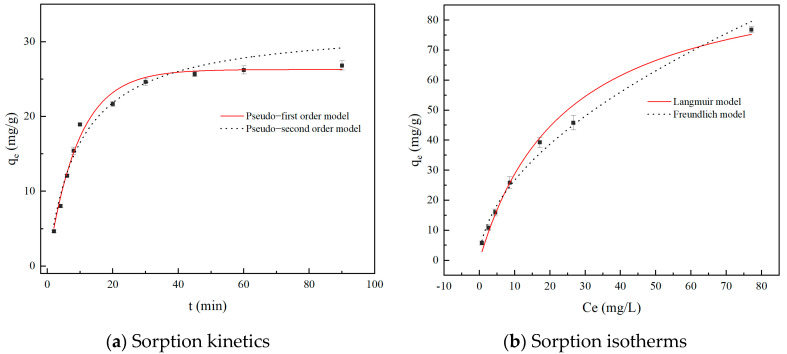
(**a**) Sorption kinetics of BCG onto CBC, and (**b**) sorption isotherms of BCG onto CBC.

Adsorption kinetics not only describes the adsorption process but also helps understand how adsorbent materials interact with adsorbate molecules. Considering the pH and temperature of real BCG wastewater, the adsorption process of 0.08 g of biochar on 50 mg/L wastewater was studied at pH = 6 and a temperature of 298 K. The pseudo-first order and pseudo-second order kinetic models were used to fit the process of CBC adsorption of BCG, as shown in Figure 5a. Analysis of the adsorption process shows that the amount of adsorption increases rapidly within 0 to 30 min. After 30 min, the adsorption amount of BCG remains relatively constant, indicating that equilibrium has been reached. This is because, after the initial acceleration phase, BCG rapidly occupies most of the active adsorption sites, causing the adsorption capacity to increase slowly with time [46].

The fitting models show good results for both, with fitting parameters listed in Table 2. The correlation coefficients (R^2^) for the pseudo-first order and pseudo-second order kinetic models are 0.9811 and 0.9610, respectively, indicating that both physical and chemical adsorption influence the adsorption process [47]. Additionally, the theoretical adsorption values obtained from the pseudo-first order kinetic model are closer to the experimental values, suggesting that this model better fits the adsorption kinetics of CBC for BCG. This is consistent with the adsorption of CBG in water by the pine cone-derived activated biochar [20].

#### 2.3.2. Adsorption Isotherm

The adsorption isotherm models describe the interactions between the adsorbent and the adsorbate, helping to determine the specific adsorption behavior of CBC for BCG. The adsorption data for BCG at 298 K were fitted using the Langmuir and Freundlich models, as shown in Figure 5b, with the parameters listed in Table 3.

As shown in Table 3, the R^2^ of the Langmuir model (0.9906) is higher than that of the Freundlich model (0.9865), indicating that the Langmuir isotherm model is more suitable for describing the adsorption process of BCG on CBC. This suggests that the adsorption of BCG on CBC follows a monolayer adsorption process, which is consistent with studies on the adsorption of BCG by rice husk biochar [19]. Additionally, the theoretical monolayer maximum adsorption capacity (q_m_) of BCG at 298 K calculated using the nonlinear Langmuir equation is 99.18 mg/g, which is significantly higher than the recently reported values of 8.75 mg/g for pine cone biochar, 37.79–45.47 mg/g for rice straw biochar, and 14.78–18.08 mg/g for rice husk biochar [19,20].

Due to the high R^2^ value (0.9865) of the Freundlich isotherm model, the results of this model are also of great reference value. As shown in Table 3, the dimensionless separation factor (R_L_) of the Langmuir equation is 0.0408, which is between 0 and 1, while the nonlinear exponent (1/n) of the Freundlich equation is 0.5347, which is less than 1. This indicates that CBC is favorable for BCG adsorption under the study conditions. Therefore, the selected biochar is a suitable adsorbent for BCG in aqueous solutions [48].

### 2.4. Possible Adsorption Mechanism

Figure 3a shows the FTIR spectrums of biochar before and after adsorption of BCG. After adsorption of CBG, the absorption peak positions of various functional groups on CBC underwent slight changes. Specifically, the –OH absorption peak shifted to 3409 cm^–1^, and the absorption peak of the C=O group in the –COOH group shifted to 1401 cm^–1^. This indicates the possible presence of hydrogen bonding interactions involving –OH and –COOH between CBC and BCG, leading to changes in their stretching vibration positions. Additionally, the absorption peak of C-H in the aromatic ring shifts to 863 cm^–1^, suggesting that the aromatic ring also participated in the adsorption process.

Figure 3d,f presented the XPS spectra of CBC after BCG adsorption. The deconvolution peak of C1s revealed that the content of C=C or C–C bonds in CBC increased by 14.23%, which indicate that a large amount of BCG was adsorbed onto the surface of CBC, leading to a higher proportion of aromatic rings. The C–O content decreased by 19.86%, suggesting the presence of hydrogen bonding between the –OH groups and BCG. The disappearance of the O-C=O peak indicates that –COOH groups are bound to BCG in the form of hydrogen bonds. The deconvolution peak of O 1s showed that the C–O content decreased by 16.95%, further confirming the hydrogen bonding interaction between the –OH groups and BCG. These results are consistent with the FTIR measurements.

### 2.5. DFT Calculations

Biochar (BC) is a highly aromatic material with a structure similar to graphite. To simplify the simulation, we use a simple graphite structure composed of twelve aromatic rings as the BC model, with its optimized configuration shown in Figure S1 [49]. The XPS and FTIR characterization results of the CBC surface indicate the presence of –OH and –COOH groups. Based on the BC simulation model, models for CBC were constructed, namely BC–OH and BC–COOH, with configurations also shown in Figure S1.

**Figure 6:**
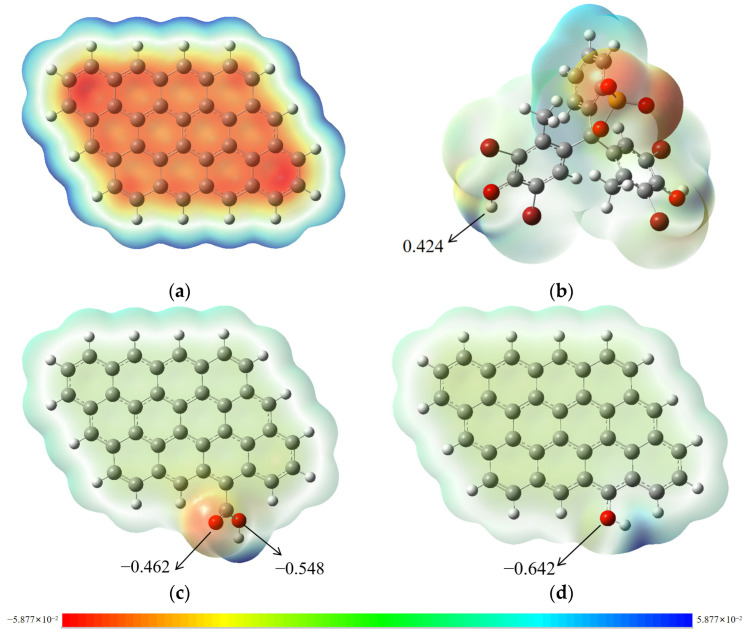
The ESP of (**a**) BC, (**b**) BCG, (**c**) BC–COOH and (**d**) BC–OH.

To understand the interaction sites between biochar and BCG, the natural population analysis (NPA) charge and surface electrostatic potential (ESP) distributions of the CBC and BCG models were calculated using the DFT theory. As shown in Figure 6, the electron density of different regions of the molecule is distinguished by color, with red indicating high electron density regions and blue indicating low electron density regions. The positive electrostatic potential of BCG is concentrated on the H atoms of the –OH groups, while the negative electrostatic potential of the biochar model is concentrated on the aromatic rings, the O atoms of –COOH, and the O atoms of –OH. Therefore, the binding sites for electrostatic interactions are likely to occur between the H atoms of the –OH groups on BCG and the aromatic rings, as well as the –OH or –COOH groups in biochar.

**Figure 7:**
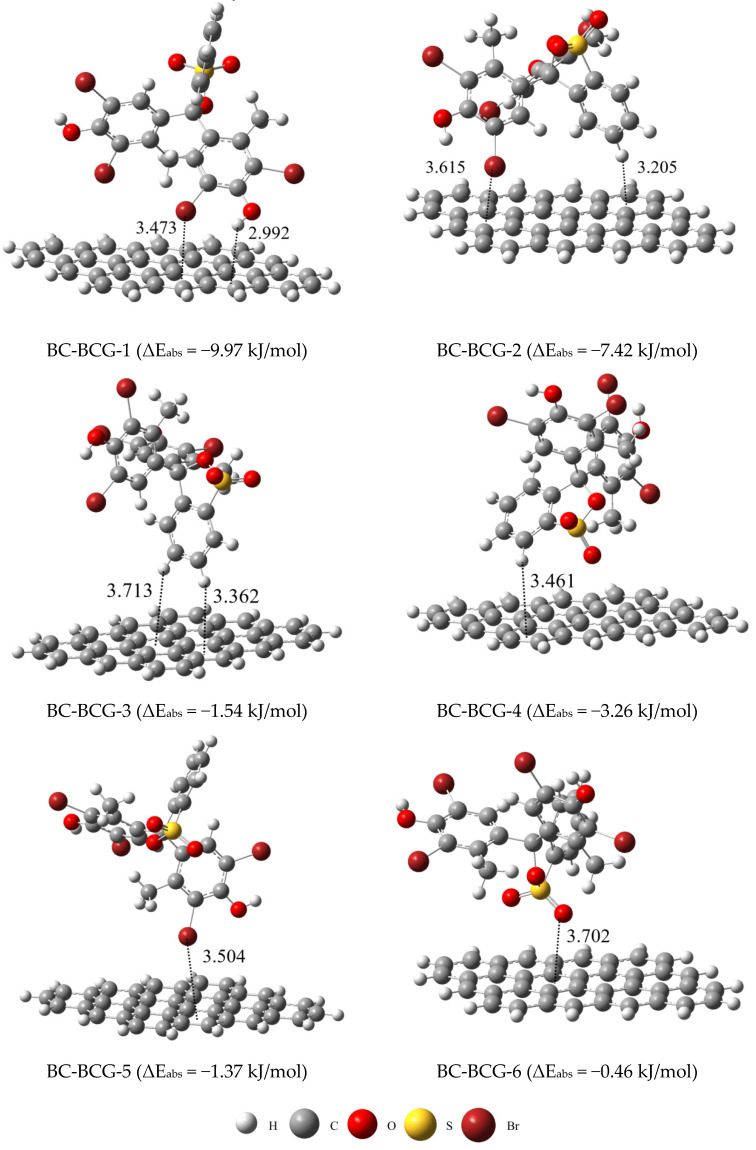
Optimal adsorption configuration and calculated adsorption energies for BCG adsorbed on BC.

Through multiple simulation experiments, we have obtained six different adsorption configurations, representing various binding characteristics. The adsorption configurations and adsorption energies are listed in Figure 7. As seen in Figure 7, the binding configuration of BC-BCG-1 shows that the oxygen groups in BCG can act as hydrogen bond donors, forming π–type hydrogen bonds with the π–electron cloud of the carbon structure in the BC [50]. The bond length is 2.992 Å, which is consistent with known intermolecular interaction bond lengths [49]. Additionally, there are interactions between the Br in BCG and the aromatic rings of BC, known as halogen–π interactions [51,52].

The binding configuration of BC-BCG-2 shows that, in addition to the halogen−π interaction between the aromatic rings of BC and BCG, π–π interactions can also occur between the aromatic rings of BC and the aromatic rings of BCG [53]. The bond length is 3.205 Å, consistent with the distance at which π-π interactions occur [54]. In the binding configurations of BC-BCG-3 and BC-BCG-4, only π–π interactions are present, while BC-BCG-5 exhibits only halogen−π interactions. Therefore, their adsorption energy is lower than that of BC-BCG-1 (−9.97 kJ/mol) and BC-BCG-2 (−7.42 kJ/mol). The binding energy of BC-BCG-6 is very low, at only −0.36 kJ/mol, indicating that its adsorption is likely controlled by weak van der Waals forces (0.4–4 kJ/mol) [49].

**Figure 8:**
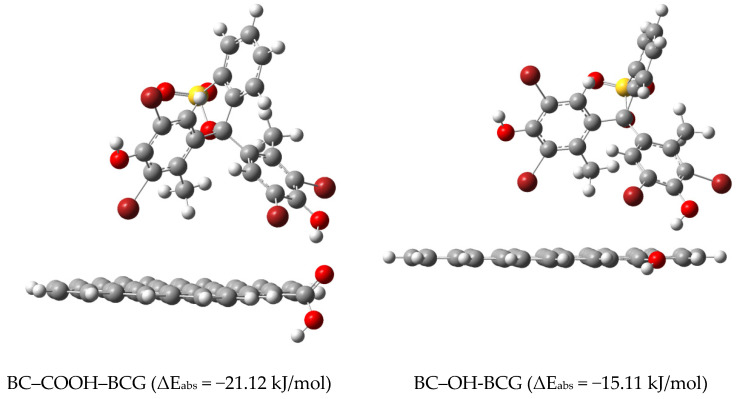
Optimal adsorption configuration and calculated adsorption energies for BCG adsorbed on BC–COOH and BC–OH.

The adsorption configurations and adsorption energies of BCG adsorbed on BC–COOH and BC–OH are listed in Figure 8. The adsorption binding energy of BC–COOH with BCG is very high (−21.12 kJ/mol), which may be due to the formation of hydrogen bonds between the –COOH groups of biochar and the H atoms in BCG, thereby enhancing intermolecular interactions [19]. Similarly, the –OH group in BC–OH can also form hydrogen bonds with the H atom of BCG, with a bond length of 1.942 Å. The adsorption energy is −15.11 kJ/mol, which is lower than that of BC–COOH with BCG. This indicates that the strength of hydrogen bond between BCG and the –COOH group is greater than that between BCG and the –OH group, consistent with Zhang’s research [49].

### 2.6. Cyclic Adsorption Experiment

**Figure 9:**
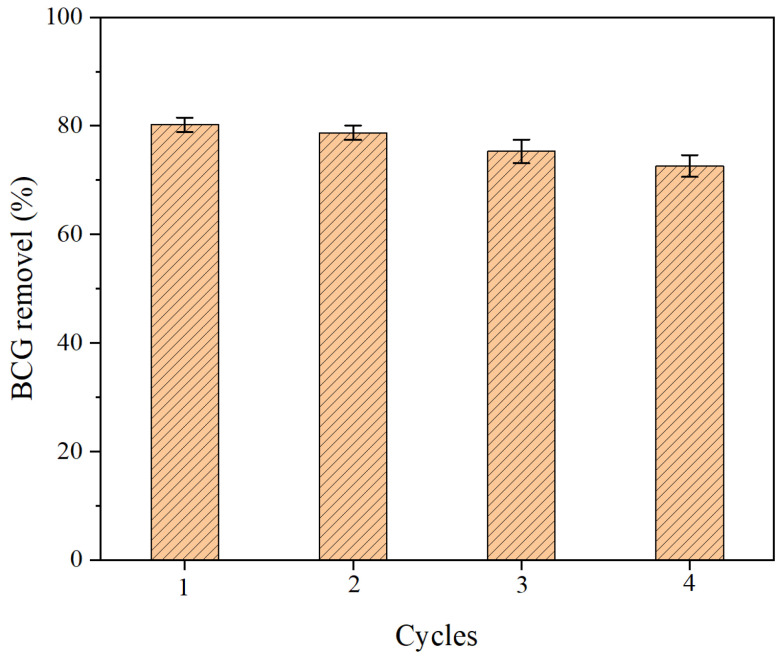
Removal rate at different cycle numbers.

To study the reusability of CBC, the adsorbent with adsorbed BCG was soaked in anhydrous ethanol for 1 h and then treated with deionized water [55]. As shown in Figure 9, four rounds of adsorption–desorption experiments were conducted at room temperature. It was observed that the adsorption performance of CBC decreased with each cycle but still maintained good performance, with removal rates above 70%. This indicates that some adsorption sites may still be occupied by BCG or residual ethanol molecules during the desorption process. The prepared CBC is a reusable adsorbent with effective removal of BCG.

## 3. Materials and Methods

### 3.1. Chemicals and Materials

Cucumber straw was sourced from a vegetable greenhouse in Shouguang City, Shandong Province. To ensure the accuracy of the experiment, it was washed three times with deionized water and then dried for use. BCG was provided by Aladdin Chemical Reagent Co., Ltd. (Shanghai, China). Sodium hydroxide (NaOH) and hydrochloric acid (HCl) were purchased from Sinopharm Chemical Reagent Co., Ltd. (Shanghai, China). All solutions were prepared using ultrapure water produced by the Millipore water purification system.

### 3.2. Preparation of CBC

The collected cucumber stalks were washed three times and then dried in a hot air oven at 353 K until a constant weight was achieved. The dried stalks were then physically crushed and screened through a 60-mesh nylon sieve. Next, the powdered straw was placed in a sealed small drawer-type carbonization furnace (THL-CTS-400 × 600). In an inert atmosphere, they were heated to 873 K at a rate of 5 K/min and maintained at this temperature for 120 min. The resulting biochar, named CBC, was cooled to room temperature. It is ground into powder and stored in a sealed bottle for future use.

### 3.3. Characterization of CBC

The experiment used a fully automatic surface area and porosity analyzer (Quadrasorb Si, Quantachrome Instruments, Boynton Beach, FL, USA) to conduct Brunauer–Emmett–Teller (BET) tests for examining pore size characteristics. The morphology of the samples was determined using a scanning electron microscope (SEM, JEM-2100, JEOL, TMD, Osaka, Japan). The surface functional groups of the biochar were analyzed using Fourier transform infrared spectroscopy (FTIR, Nicolet iS50, Thermo Nicolet, Waltham, MA, USA) over a wavenumber range of 400–4000 cm^–1^. The crystalline structure of the biochar was determined using X-ray diffraction analysis (XRD, Bruker D8 Advance, SL, Germany). X-ray photoelectron spectroscopy (XPS, Thermo Scientific K-Alpha, Waltham, MA, USA) was employed to analyze and test the types of functional groups containing elements such as C and O on the surface of the sample.

### 3.4. Batch Adsorption Experiments for BCG Solutions

Adsorption experiments were conducted using a batch process. A 50 mL solution of BCG at a concentration of 50 mg/L was added to a conical flask. The pH of the BCG solution was adjusted using 0.1 M NaOH and 0.1 M HCl solutions. Under constant stirring conditions at 220 rpm in a water bath, the effects of various parameters on the adsorption process were investigated, including initial pH (2, 4, 6, 8, 10), adsorbent dosage (0.02 g, 0.04 g, 0.06 g, 0.08 g, 0.10 g), and temperature (298 K, 313 K, 323 K, 333 K, 353 K). All adsorption experiments were repeated three times, and the results were averaged to ensure accuracy and reliability. After 60 min, 2.0 mL of supernatant was collected and passed through a 0.22 μm microporous filter membrane. The initial concentration and residual concentration of BCG were measured at a wavelength of 424 nm using a UV–Vis spectrophotometer (UV-3600, Shimadzu, Japan) [56]. The removal efficiency of BCG was calculated using equation (1), while the adsorption amount at any time (q_t_, mg/g) and the adsorption amount at equilibrium (q_e_, mg/g) were calculated using Equations (2) and (3), respectively [57].

(1) $$Removal(\%)=\frac{C_{0}-C_{t}}{C_{0}}\times 100\%$$

(2) $$q_{t}=\frac{(C_{0}-C_{t})\times V}{m}$$

(3) $$q_{e}=\frac{(C_{0}-C_{e})\times V}{m}$$

where Removal (%) is associated with the removal efficiency of BCG; C_0_ (mg/L) is the initial BCG concentration; C_t_ (mg/L) is the BCG concentration at any time t; C_e_ (mg/L) is the equilibrium BCG concentration; V (mL) is the volume of the BCG solution and m (g) represents the mass of the adsorbent dose.

### 3.5. Adsorption Models

The adsorption kinetics experiments were conducted in 250 mL conical flasks, with 50 mL of a 50 mg/L BCG solution adjusted to pH=6. The amount of biochar added was 0.08 g, and the stirring speed was maintained at 220 rpm in a water bath at 298 K. After the experiment started, samples were taken at 2, 4, 6, 8, 10, 20, 30, 45, 60, and 90 min. The samples were filtered through a 0.22 μm microporous filter membrane to determine the solution concentration. The adsorption characteristics during the reaction process were studied using pseudo-first order and pseudo-second order kinetic models, with the simulation Formulas (4) and (5), respectively [58].

q_t_ = q_e_(1 − e^−k1t^) (4)

q_t_ = q_e_^2^k_2_t/(1 + q_e_k_2_t) (5)

where t (min) is the adsorption time; k_1_ and k_2_ are the reaction rate constants for pseudo-first order and pseudo-second order kinetics, with units of min^−1^ and mg·g^−1^·min^−1^, respectively.

For the equilibrium study and construction of the adsorption isotherm of BCG by CBC, different mass concentrations (10, 20, 30, 50, 80, 100, 200 mg/L) of BCG solutions were prepared. Adjust the pH to 6, add 0.08 g of biochar, and maintain a stirring speed of 220 rpm in a water bath at 298 K. After 60 min, collect samples to determine the residual concentration of BCG in the solution. Then, Langmuir and Freundlich models were used to analyze the obtained adsorption isotherm data, and the model formulas were fitted with simulation Formulas (6) and (7), respectively.

(6) $$q_{e}=\frac{q_{m}k_{L}C_{e}}{1+k_{L}C_{e}}$$

(7) $$q_{e}=k_{F}{C_{e}}^{\frac{1}{n}}$$

where q_m_ (mg/g) is the maximum of adsorption capacity, k_L_ (L/mg) represents the constant of Langmuir model, k_F_ (mg g^−1^(L mg^−1^) 1/n) is the constant of Freundlich model, and 1/n stands for a constant that describes the heterogeneity of the adsorbent surface.

### 3.6. DFT Calculation

DFT calculations were performed using the Gaussian16 program [59]. Geometry optimization and frequency calculations were conducted at the B3lyp/6-31G(d) level. Solvent effects of water were examined using the self-consistent reaction field (SCRF) method based on the SMD model [60]. The adsorption energy calculation formula is as follows:

ΔE_ads_ = E_total_ − (E_biochar_ + E_BCG_) (8)

where E_total_ is the total energy after adsorption, E_biochar_ is the energy of biochar, and E_BCG_ is the energy of BCG.

## 4. Conclusions

This article reports on the influencing factors and adsorption characteristics of BCG adsorption by CBC, as well as the mechanism of the interface interaction between BCG and CBC studied through DFT calculations. The characterization results indicate that the surface of CBC has a graphitized structure and contains a large number of active functional groups, such as −OH and −COOH. Adsorption experiments show that low pH and high temperature are favorable for the adsorption of BCG. The adsorption process is controlled by both physical and chemical interactions. The adsorption isotherm fits well with the Langmuir model, indicating that the adsorption is monolayer adsorption. Experimental and DFT calculation results demonstrate that there are electrostatic interactions, van der Waals forces, halogen−π interactions, π–π interactions, and hydrogen bonding interactions between CBC and BCG, with the hydrogen bond between BCG and –COOH group being stronger than that formed with the –OH group. This result reveals the intermolecular reactions involved in the adsorption and removal of BCG from a mechanistic perspective, providing important reference value for effectively removing organic pollutants from wastewater using crop straw waste.

## Figures and Tables

**Table 1:** Physical characteristics of CBC.

Materials	BET Specific Surface Area (m^2^/g)	Total Pore Volume (cm^3^/g)	Average Pore Size (nm)	C (%)	H (%)	O (%)	N (%)
CBC	101.58	0.0942	1.85	71.23	3.12	25.19%	0.27

**Table 2:** Parameters within different sorption kinetic models for adsorption of BCG onto CBC.

Kinetic Models	Parameters	Value
Pseudo-first order model	q_e_ (mg/g)	26.28
	k_1_ (min^−1^)	0.1072
	R^2^	0.9811
Pseudo-second order model	q_e_ (mg/g)	32.26
	k_2_ (mg·g^−1^·min^−1^)	3.25 × 10^−3^
	R^2^	0.9610

**Table 3:** Parameters within different sorption isotherms models for adsorption of BCG onto CBC.

Isotherm Models	Parameters	Value
Langmuir model	q_m_ (mg/g)	99.18
	K_L_ (L/mg)	0.0408
	R^2^	0.9906
Freundlich model	K_f_ (mg/g·(L/mg)^1/n^)	7.79
	1/n	0.5347
	R^2^	0.9865

## Data Availability

Data are contained within the article and Supplementary Materials.

## Supplementary Materials

The following supporting information can be downloaded at: https://www.mdpi.com/article/10.3390/molecules29194517/s1. Figure S1: Optimized configurations of BC, BCG, BC–COOH, and BC–OH; Table S1: Cartesian coordinates for all the optimized structures.
