# Supplementary material for: Experimental and Theoretical Studies on the Adsorption of Bromocresol Green from Aqueous Solution Using Cucumber Straw Biochar
Source: Molecules. 2024 Sep 24;29(19):4517. doi: 10.3390/molecules29194517 (PMC11477533; doi:10.3390/molecules29194517)
Supplement: Supplementary file 1 [file molecules-29-04517-s001.zip › molecules-3187128-supplementary.pdf]

Supplementary information for

Experimental and theoretical studies on the adsorption of bromocresol green from aqueous solution using cucumber straw biochar

Chenxi Zhang[1], Lingbin Meng[1], Zhihao Fang[1,*], Youxin Xu[1], Yue Zhou[1], Hongsen Guo[1], Jinyu Wang[1], Xiaotian Zhao[1], Shuyan Zang[2] and Hailin Shen[3,*]

[1] Shandong Provincial University Laboratory for Protected Horticulture, Weifang University of Science and Technology, Weifang 262700, China; morningsunzhang@outlook.com (C.Z.); mlb8124@126.com(L.M.); xuyouxin@wfust.edu.cn (Y.X.); 1695575319@qq.com (Y.Z.); g2037@outlook.com (H.G.); jinyu678678@outlook.com (J.W.); ZhaoXiaotian0520@outlook.com (X.Z.);

[2] College of Science, Shenyang University of Chemical Technology, Shenyang 110142, China; zangshuyan@126.com (S.Z.)

[3] School of Chemical Engineering and Materials, Changzhou Institute of Technology, Changzhou 213032, China

* Correspondence: wfsgfzh@163.com (Z.F.); 18721967108@163.com (H.S.)

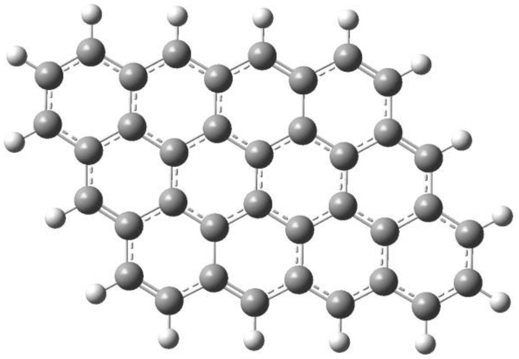	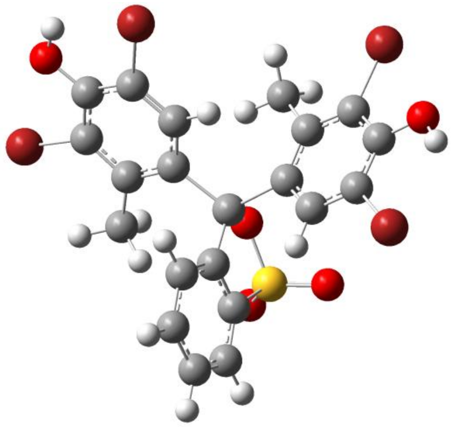
BC	BCG
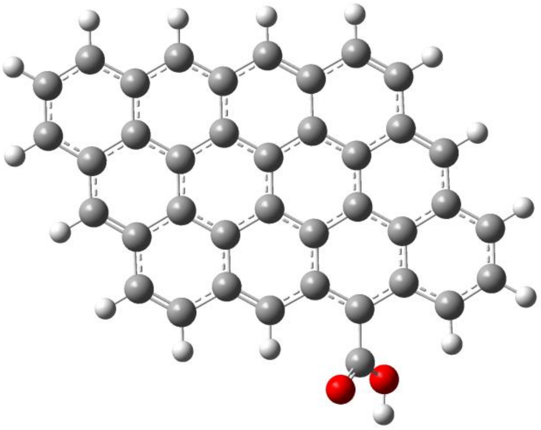	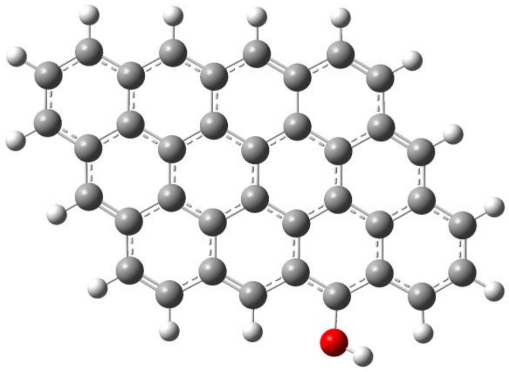
BC-COOH	BC-OH

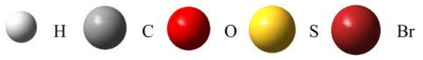

Figure S1. Optimized configurations of BC, BCG, BC-COOH and BC-OH

Table S1. Cartesian coordinates for all the optimized structures

BC

C	-2.13960500	1.40377900	0.00000000
C	-0.77139200	1.72380400	0.00000000
C	0.20724400	0.68419500	0.00000000
C	-0.20724400	-0.68419500	0.00000000
C	-1.58561900	-1.00399600	0.00000000
C	-2.55711300	0.03513300	0.00000000
C	1.58561900	1.00399600	0.00000000
C	0.77139200	-1.72380400	0.00000000
C	2.13960500	-1.40377900	0.00000000
C	2.55711300	-0.03513300	0.00000000
C	3.13729800	-2.45073300	0.00000000
C	2.68057800	-3.82412300	0.00000000
C	1.35991200	-4.13393900	0.00000000
C	0.34026200	-3.10682400	0.00000000
C	-1.00591800	-3.40451900	0.00000000
C	-2.00803400	-2.38409700	0.00000000
H	-1.32599400	-4.44368900	0.00000000
H	3.43147500	-4.61003800	0.00000000
H	1.03626800	-5.17153000	0.00000000
C	-0.34026200	3.10682400	0.00000000
C	1.00591800	3.40451900	0.00000000
C	2.00803400	2.38409700	0.00000000
H	1.32599400	4.44368900	0.00000000
C	3.36995800	2.67844700	0.00000000
C	4.35416300	1.65994000	0.00000000
C	3.93923500	0.28644900	0.00000000
H	3.69058200	3.71732400	0.00000000
C	4.47673100	-2.11839500	0.00000000
H	5.22489700	-2.90758900	0.00000000
C	4.91867100	-0.76026200	0.00000000

C	-3.36995800	-2.67844700	0.00000000
C	-4.35416300	-1.65994000	0.00000000
C	-3.93923500	-0.28644900	0.00000000
C	-4.91867100	0.76026200	0.00000000
C	-5.74061800	-1.94990900	0.00000000
C	-6.68009800	-0.92661600	0.00000000
H	-7.73916100	-1.16894500	0.00000000
C	-6.28156100	0.41506800	0.00000000
C	-3.13729800	2.45073300	0.00000000
C	-1.35991200	4.13393900	0.00000000
C	5.74061800	1.94990900	0.00000000
C	6.28156100	-0.41506800	0.00000000
C	6.68009800	0.92661600	0.00000000
H	7.73916100	1.16894500	0.00000000
H	-3.69058200	-3.71732400	0.00000000
H	-6.05967800	-2.98865800	0.00000000
H	-7.02693300	1.20582100	0.00000000
H	-1.03626800	5.17153000	0.00000000
H	6.05967800	2.98865800	0.00000000
H	7.02693300	-1.20582100	0.00000000
C	-2.68057800	3.82412300	0.00000000
C	-4.47673100	2.11839500	0.00000000
H	-3.43147500	4.61003800	0.00000000
H	-5.22489700	2.90758900	0.00000000

BCG

C	-0.15335200	1.07565500	0.20334100
C	1.17425700	0.28723800	0.19076200
C	2.09722400	0.51527100	-0.83237100
C	1.48558200	-0.64336600	1.21723400
C	3.31515800	-0.14703900	-0.85791900
H	1.87496700	1.21431200	-1.62719500
C	2.73402400	-1.27781200	1.16182800
C	3.67226800	-1.05575600	0.14135100
C	-1.39808200	0.19251700	0.00314800
C	-1.19702700	-1.04939600	-0.60610400
C	-2.71651900	0.60315900	0.33702100
C	-2.25695500	-1.90514200	-0.85656600
H	-0.20152700	-1.35911200	-0.89372800
C	-3.75570500	-0.31180300	0.10294000
C	-3.56280200	-1.56945200	-0.49310500
C	-0.14451000	2.26545700	-0.76273200
C	0.17698000	3.47799800	-0.16137900
C	-0.41263500	2.24476600	-2.13003400
C	0.26841500	4.68154700	-0.85288100
C	-0.32947300	3.43972400	-2.85059200
H	-0.68281000	1.31718000	-2.62452300
C	0.01146700	4.64468400	-2.22413400
H	0.51335200	5.61044600	-0.34837000
H	-0.54017000	3.43182000	-3.91544000
H	0.06408600	5.56060700	-2.80363200
O	-0.23664100	1.71597200	1.55012300
S	0.40178500	3.23134700	1.57567400
O	1.81965600	3.17493400	1.94968600
O	-0.48579800	4.05319900	2.40257600
O	-4.63025300	-2.38081700	-0.68810000
H	-4.33338800	-3.20359100	-1.12204900
Br	-5.55907700	0.10248500	0.60261400

Br	-1.93360600	-3.59768200	-1.69249800
C	-3.03104900	1.95927100	0.92129700
H	-2.92905900	1.95819300	2.01204900
H	-2.37643100	2.73286300	0.52330500
H	-4.05623200	2.24879300	0.68762100
Br	4.55042800	0.19159100	-2.28223200
Br	3.23302400	-2.52137400	2.52683300
O	4.85226200	-1.72023500	0.17348800
H	5.38904300	-1.46258700	-0.60041900
C	0.54964400	-0.98673300	2.35133600
H	0.40101600	-2.07043000	2.40246500
H	0.98153200	-0.67770300	3.31031400
H	-0.42511300	-0.51723200	2.25687300

BC-COOH

C	2.25164200	-1.45369100	-0.00783000
C	0.84798800	-1.52008700	-0.01641700
C	0.06556400	-0.32759200	-0.01594700
C	0.72303300	0.94292700	-0.00302600
C	2.13716700	1.00990100	0.00369500
C	2.90822900	-0.18439800	0.00161800
C	-1.35080600	-0.39691900	-0.02256300
C	-0.04895000	2.14193000	0.00044300
C	-1.45303400	2.07453900	-0.00443700
C	-2.11579000	0.80420400	-0.01405200
C	-2.23640100	3.28936700	-0.00387400
C	-1.53938100	4.55731300	0.00634800
C	-0.18553200	4.62183800	0.01344700
C	0.62612900	3.42475000	0.01052400
C	2.00340900	3.47453500	0.01694100
C	2.80215900	2.29040700	0.01409400
H	2.50585300	4.43857300	0.02475200
H	-2.13705800	5.46506100	0.00752400
H	0.32395000	5.58172700	0.02096300
C	0.17853800	-2.79872400	-0.02160700
C	-1.19866600	-2.85590600	-0.02920000
C	-2.01504700	-1.68084800	-0.03900500
H	-1.67177700	-3.83022900	-0.03197400
C	-3.42318000	-1.72465900	-0.01879300
C	-4.20822600	-0.52648400	-0.02000400
C	-3.53531400	0.74272300	-0.01854600
C	-3.61166400	3.21065200	-0.01730000
H	-4.20245400	4.12342000	-0.02161700
C	-4.29696900	1.95990100	-0.02904000
C	4.19502000	2.33606300	0.02129500
C	4.97997700	1.15824400	0.01931300
C	4.32454300	-0.11692800	0.00976500

C	5.09871000	-1.32180600	0.00902000
C	6.39557000	1.19504900	0.02694300
C	7.13605200	0.01969600	0.02540500
H	8.22115800	0.06754700	0.03120100
C	6.50156700	-1.22756500	0.01668000
C	3.04261100	-2.66353100	-0.00548600
C	0.99183100	-3.99547400	-0.01601500
C	-5.62535500	-0.52759100	-0.07118500
C	-5.69957100	1.89497300	-0.05728200
C	-6.34635400	0.65942500	-0.08615700
H	-7.43140500	0.62078900	-0.12311000
H	4.69584900	3.30086300	0.02905300
H	6.89600300	2.15941400	0.03414400
H	7.09217400	-2.13961700	0.01601500
H	0.48301800	-4.95570200	-0.01868100
H	-6.16216400	-1.46778600	-0.10030700
H	-6.27281000	2.81779400	-0.06524100
C	2.34677200	-3.93188700	-0.00960500
C	4.42055900	-2.57808900	0.00191000
H	2.94325700	-4.84036900	-0.00687400
H	5.01444900	-3.48889200	0.00318200
C	-4.09639000	-3.05642100	-0.01864100
O	-4.96467000	-3.21361700	1.00575000
H	-5.37755500	-4.09633500	0.90891000
O	-3.89322200	-3.94579600	-0.83281600

BC-OH

C	-2.18282900	-1.40687200	-0.00213400
C	-0.79722300	-1.64538300	0.00245400
C	0.12042000	-0.55157900	0.00359200
C	-0.37534500	0.79049700	0.00500000
C	-1.77073300	1.02838300	0.00243700
C	-2.68041500	-0.06571000	-0.00296800
C	1.51715300	-0.78993700	0.00411100
C	0.53894100	1.88626300	0.01058300
C	1.92363500	1.64647600	0.01063400
C	2.42257900	0.30670600	0.00675500
C	2.85500000	2.75217300	0.01282300
C	2.31871500	4.09667700	0.02050800
C	0.98214500	4.32741400	0.02141700
C	0.02649300	3.24047100	0.01474600
C	-1.33481000	3.45866100	0.01218800
C	-2.27503300	2.38107000	0.00504100
H	-1.71469600	4.47752700	0.01545600
H	3.02232500	4.92515500	0.02448600
H	0.59748400	5.34404900	0.02653700
C	-0.28665000	-2.99884400	0.00954800
C	1.07520500	-3.21894200	0.01856600
C	2.01071300	-2.14156500	0.01249900
H	1.44558200	-4.23840600	0.02753300
C	3.39674800	-2.36110800	0.01960800
C	4.32962500	-1.27955700	-0.00817400
C	3.82001000	0.06279300	-0.00100100
C	4.21066000	2.49886400	0.00352300
H	4.91244400	3.32940600	0.00169600
C	4.73217400	1.16993500	-0.00838900
C	-3.65194800	2.59525700	0.00036200
C	-4.57539600	1.52142000	-0.00657300
C	-4.07970500	0.17406000	-0.00762700

C	-4.99589200	-0.92944200	-0.01316100
C	-5.97672100	1.72883600	-0.01226000
C	-6.85329300	0.65107200	-0.01858400
H	-7.92501600	0.83064100	-0.02303100
C	-6.37730400	-0.66474400	-0.01894700
C	-3.11641200	-2.51080500	-0.00521900
C	-1.24159500	-4.08514300	0.00794100
C	5.73092900	-1.47304600	-0.05128600
C	6.11471600	0.91910700	-0.03306700
C	6.60065900	-0.38938800	-0.06080500
H	7.67178100	-0.56556300	-0.09355200
H	-4.03173100	3.61406200	0.00190300
H	-6.35662200	2.74693100	-0.01158700
H	-7.07495000	-1.49798200	-0.02342600
H	-0.85667900	-5.10171200	0.01254800
H	6.15382700	-2.47261200	-0.09277300
H	6.80406300	1.75898500	-0.03843600
C	-2.57863800	-3.85458000	-0.00036100
C	-4.47370200	-2.25860400	-0.01159200
H	-3.28108700	-4.68414900	-0.00260900
H	-5.17316300	-3.09133200	-0.01460300
O	3.80379400	-3.66425200	0.04116000
H	4.75883900	-3.72629600	0.20745500

BC-BCG-1

C	-3.36242400	-1.39862400	0.18491400
C	-3.38095600	0.14143000	0.09271000
C	-4.31758600	0.76555300	-0.73416900
C	-2.47411300	0.93613600	0.84348500
C	-4.37150500	2.14739700	-0.83710300
H	-5.01869300	0.17919400	-1.31252700
C	-2.55538000	2.32837300	0.70244800
C	-3.49177300	2.96716300	-0.12631000
C	-2.03983900	-2.02660000	-0.29194300
C	-1.24330700	-1.25261400	-1.14122000
C	-1.62905000	-3.34477600	0.04137800
C	-0.03286200	-1.72554400	-1.62039100
H	-1.56341600	-0.26162000	-1.43362400
C	-0.37700300	-3.76927000	-0.43236600
C	0.44742900	-2.98622200	-1.25729800
C	-4.57172700	-2.05261600	-0.49026500
C	-5.59815700	-2.38708400	0.38662100
C	-4.72865300	-2.33318700	-1.84643000
C	-6.79078900	-2.98162100	-0.01342100
C	-5.91697400	-2.92982400	-2.27718600
H	-3.93913400	-2.09663600	-2.55240600
C	-6.93921600	-3.24911200	-1.37489000
H	-7.56612200	-3.23940600	0.70037600
H	-6.04466400	-3.15549200	-3.33133000
H	-7.84980600	-3.71910600	-1.73194100
O	-3.54451500	-1.72634800	1.63316300
S	-5.11804500	-1.97645100	2.03867200
O	-5.72543300	-0.72168800	2.49645000
O	-5.14673600	-3.11874400	2.95630800
O	1.64042000	-3.48108000	-1.66222500
H	2.11196900	-2.80804400	-2.19097900
Br	0.31408700	-5.49362000	0.04054100

Br	1.03931100	-0.62499100	-2.75882100
C	-2.47065200	-4.28544900	0.86966000
H	-2.27951900	-4.15784100	1.94091500
H	-3.53479200	-4.13496300	0.69749400
H	-2.24367100	-5.32229300	0.61770200
Br	-5.67742900	2.96341100	-1.97738200
Br	-1.32768200	3.45179200	1.64570200
O	-3.49663800	4.32044600	-0.19700000
H	-4.19857300	4.60702500	-0.81217900
C	-1.42683600	0.36669000	1.77064600
H	-0.42578900	0.67597400	1.45090600
H	-1.57027000	0.75536700	2.78506200
H	-1.44607300	-0.71797200	1.82251900
C	3.78383000	-1.10055600	1.76349300
C	3.34736600	0.20713900	2.03496100
C	3.31215500	1.18141700	0.99172700
C	3.72608200	0.82479700	-0.32972600
C	4.16710200	-0.49186900	-0.60017100
C	4.19772300	-1.46013200	0.44151900
C	2.87181500	2.49860900	1.26294500
C	3.69473100	1.80017500	-1.37154300
C	3.25822100	3.10765000	-1.09994500
C	2.84026200	3.46645600	0.22093700
C	3.22569800	4.10234500	-2.14922800
C	3.65748400	3.70755900	-3.47319100
C	4.08098500	2.44597300	-3.73609200
C	4.12170600	1.43013200	-2.70578700
C	4.54966700	0.14329400	-2.95621000
C	4.58947300	-0.85449400	-1.93204000
H	4.86882000	-0.13094500	-3.95886400
H	3.63387200	4.45692600	-4.26024800
H	4.40192300	2.16877900	-4.73694200
C	2.92707300	0.57947900	3.37060300
C	2.49933200	1.86626500	3.62043300

C	2.45337800	2.86205300	2.59547900
H	2.18489400	2.14185200	4.62423100
C	2.01838500	4.16288900	2.84117900
C	1.98008600	5.14267400	1.81871000
C	2.39829600	4.78673700	0.49316100
H	1.70361500	4.43883700	3.84469900
C	2.79277300	5.38154600	-1.86525300
H	2.76989500	6.12765300	-2.65610300
C	2.37034200	5.76352600	-0.55535000
C	5.01415700	-2.15869000	-2.17965800
C	5.05301600	-3.13893100	-1.15680300
C	4.63818800	-2.78144800	0.16960800
C	4.66887800	-3.75711800	1.21925700
C	5.48850000	-4.46467000	-1.39781500
C	5.51168800	-5.40164500	-0.37227200
H	5.84833500	-6.41414300	-0.57691800
C	5.10867300	-5.05938700	0.92331800
C	3.82083400	-2.09343000	2.81421500
C	2.97457000	-0.43394900	4.40271300
C	1.54291200	6.46815300	2.05972000
C	1.92957200	7.06536400	-0.25943700
C	1.52293100	7.40646400	1.03555300
H	1.18587200	8.41877300	1.24037100
H	5.33305500	-2.43387400	-3.18209100
H	5.80367300	-4.73956800	-2.40076700
H	5.13276800	-5.80171900	1.71657800
H	2.65943200	-0.15472000	5.40482900
H	1.22511400	6.74193900	3.06217800
H	1.90817600	7.80869900	-1.05183900
C	3.39631300	-1.69611500	4.13975600
C	4.25195900	-3.37324600	2.53021100
H	3.42434900	-2.44399400	4.92807000
H	4.27792300	-4.11820100	3.32203400

BC-BCG-2

C	2.18493500	1.18660500	-1.65773400
C	2.37566500	-0.34373000	-1.74291800
C	1.25126900	-1.16828400	-1.82404900
C	3.67035200	-0.92648400	-1.75453300
C	1.37651400	-2.54539100	-1.92494500
H	0.25647000	-0.74472600	-1.80442000
C	3.75360800	-2.31910700	-1.89265200
C	2.63107000	-3.15813800	-1.97521800
C	2.58772800	1.78539300	-0.29769600
C	2.64228500	0.90779300	0.78895900
C	2.83215500	3.17020900	-0.09218900
C	2.96282200	1.35739000	2.05955100
H	2.42919400	-0.14344000	0.64997700
C	3.19306800	3.57599800	1.20259200
C	3.26332000	2.69985000	2.29879700
C	0.77831000	1.62744700	-2.08021000
C	0.69185500	2.03746500	-3.40691800
C	-0.37300200	1.65017000	-1.29515900
C	-0.48919000	2.45746200	-4.00997700
C	-1.57494000	2.06658600	-1.87538100
H	-0.33650800	1.35177900	-0.25249100
C	-1.63720300	2.46223000	-3.21695600
H	-0.51535300	2.78164600	-5.04507900
H	-2.47478400	2.08990900	-1.26906600
H	-2.58087100	2.78758400	-3.64260000
O	3.06666200	1.77534900	-2.70958900
S	2.29616100	1.97810800	-4.14726500
O	2.47848900	0.78931300	-4.98834200
O	2.73582000	3.26659500	-4.68926500
O	3.60788100	3.18693900	3.51555400
H	3.60021300	2.45775100	4.16548300
Br	3.63218200	5.40414900	1.57422600

Br	3.01223000	0.11933400	3.51920200
C	2.71840200	4.19433600	-1.19566100
H	3.63951400	4.25207000	-1.78548100
H	1.89861900	3.96397100	-1.87514700
H	2.52952900	5.18520000	-0.78175400
Br	-0.19533700	-3.63769200	-1.98147600
Br	5.46556100	-3.17030900	-1.97724100
O	2.81092600	-4.49641600	-2.09258300
H	1.93815900	-4.93345900	-2.12503100
C	4.94723400	-0.13120200	-1.62309500
H	5.60534200	-0.59528900	-0.88257000
H	5.49328400	-0.11653800	-2.57396600
H	4.77495800	0.89858700	-1.32177400
C	-2.35806600	-2.99294700	1.39646000
C	-3.31679200	-2.44971000	0.52459400
C	-3.71734000	-1.08610900	0.66027700
C	-3.13644100	-0.27169100	1.68228700
C	-2.16631200	-0.81912500	2.55458800
C	-1.77395700	-2.17962100	2.41862400
C	-4.68662500	-0.53823700	-0.21282700
C	-3.53751800	1.09187700	1.81910700
C	-4.50261300	1.63290500	0.95271400
C	-5.08472900	0.82032500	-0.07201700
C	-4.91568800	3.01267200	1.08556300
C	-4.30636700	3.80611600	2.13180000
C	-3.37371800	3.28504200	2.96770500
C	-2.94079000	1.90849600	2.85668400
C	-1.99244900	1.36387100	3.69609600
C	-1.57165400	0.00176600	3.58222500
H	-1.54511800	1.98032700	4.47219600
H	-4.61875700	4.84244300	2.23117200
H	-2.92664700	3.89734700	3.74663500
C	-3.90915400	-3.26438000	-0.51668700
C	-4.85137100	-2.71777100	-1.36170700

C	-5.27329800	-1.35598300	-1.24750400
H	-5.29389500	-3.33265700	-2.14177400
C	-6.22584000	-0.79777300	-2.09757900
C	-6.63656000	0.55217100	-1.97556300
C	-6.05665700	1.36921800	-0.94821200
H	-6.66895400	-1.41223000	-2.87749500
C	-5.86358500	3.52796500	0.22508100
H	-6.17307100	4.56535500	0.32754100
C	-6.45789100	2.73816500	-0.80614400
C	-0.61313400	-0.55326900	4.42697700
C	-0.21159200	-1.90649000	4.31284100
C	-0.80238100	-2.72781900	3.29567300
C	-0.40974000	-4.09993500	3.16144800
C	0.76256200	-2.47935900	5.16680900
C	1.13295300	-3.81117900	5.02807900
H	1.87950400	-4.23375600	5.69481400
C	0.55780100	-4.61833500	4.04009200
C	-1.95196800	-4.37476100	1.27020400
C	-3.47985300	-4.64261500	-0.62464500
C	-7.60380300	1.12815200	-2.83526100
C	-7.42262600	3.25780700	-1.68688600
C	-7.98396400	2.45596900	-2.68726000
H	-8.72758900	2.87993400	-3.35638000
H	-0.16050200	0.06605700	5.19697900
H	1.21023700	-1.86314300	5.94205400
H	0.85771700	-5.65817900	3.94215600
H	-3.92459800	-5.25433300	-1.40537900
H	-8.04560500	0.51351400	-3.61489700
H	-7.72977800	4.29487000	-1.58218500
C	-2.55761700	-5.16761100	0.22138000
C	-1.01107700	-4.89243900	2.13650200
H	-2.25074000	-6.20604700	0.12721000
H	-0.70743600	-5.93211400	2.03965600

BC-BCG-3

0 1

C	-2.98789000	-0.61848000	-1.02198300
C	-3.79659000	0.69654900	-0.99806800
C	-3.12286200	1.91086700	-0.85081900
C	-5.20817500	0.69626600	-1.15441300
C	-3.80850800	3.11584200	-0.86064800
H	-2.04899200	1.93187300	-0.72569600
C	-5.86031500	1.93682200	-1.16915300
C	-5.19447100	3.16498700	-1.02786200
C	-3.29848000	-1.55731100	0.15801200
C	-3.86143100	-0.97483600	1.29731600
C	-2.98297500	-2.94240200	0.16435600
C	-4.15517700	-1.73247300	2.41921100
H	-4.07459900	0.08549500	1.31780200
C	-3.33947800	-3.68035000	1.30447400
C	-3.92344400	-3.10937300	2.44758900
C	-1.48006900	-0.38873600	-1.17526500
C	-1.02063900	-0.50569200	-2.48319200
C	-0.56520000	-0.09033100	-0.16702000
C	0.30859000	-0.32658300	-2.85262800
C	0.77724200	0.09595100	-0.50916800
H	-0.89046900	-0.00947700	0.86515600
C	1.21212600	-0.01659300	-1.83536600
H	0.63359400	-0.43808300	-3.88192200
H	1.49724500	0.32205000	0.27095200
H	2.26080700	0.12475600	-2.07550500
O	-3.37009600	-1.30953500	-2.29071400
S	-2.36705400	-0.94597900	-3.54215800
O	-2.88644600	0.20760400	-4.28538600
O	-2.14547000	-2.18910900	-4.28554900
O	-4.22354700	-3.90269100	3.50430400
H	-4.61297800	-3.35912600	4.21594000

Br	-3.04843300	-5.57297800	1.37649500
Br	-4.91902400	-0.88944200	3.95956300
C	-2.29122000	-3.63405600	-0.98548500
H	-3.00990000	-3.97468600	-1.73891300
H	-1.57207000	-2.98087600	-1.47658700
H	-1.74696500	-4.51018500	-0.63111800
Br	-2.84325800	4.75723100	-0.65311800
Br	-7.75863300	2.01313800	-1.39131700
O	-5.91188400	4.31398000	-1.05733100
H	-5.30644800	5.07315400	-0.95447700
C	-6.03763200	-0.55757500	-1.29776800
H	-6.81428100	-0.58589700	-0.52607500
H	-6.55018300	-0.56673800	-2.26650200
H	-5.45157900	-1.46890800	-1.22181200
C	5.06618100	2.88542800	-0.10389400
C	5.31513900	1.76345500	-0.91204100
C	5.23805400	0.45000000	-0.35750800
C	4.90578000	0.27825800	1.02304000
C	4.65451200	1.40986100	1.83457500
C	4.73368500	2.71652000	1.27782000
C	5.48882000	-0.68159400	-1.16912200
C	4.82912400	-1.03515700	1.57770200
C	5.07943900	-2.15704600	0.76982500
C	5.41142300	-1.98817200	-0.61198000
C	5.00260000	-3.49061500	1.32355800
C	4.66359700	-3.62585400	2.72424300
C	4.42270600	-2.54321400	3.50535800
C	4.49186900	-1.19738100	2.97744800
C	4.24896300	-0.08643300	3.75689500
C	4.31928700	1.24071900	3.22847300
H	3.99669300	-0.21271700	4.80702000
H	4.60577100	-4.62897900	3.13884700
H	4.17009100	-2.66575500	4.55541200
C	5.65076700	1.92573100	-2.31228000

C	5.89201000	0.81464800	-3.09222100
C	5.82325500	-0.51250600	-2.56331300
H	6.14321100	0.94081600	-4.14260900
C	6.06672100	-1.63923100	-3.34592500
C	5.99452500	-2.94901800	-2.81206400
C	5.66255600	-3.12219600	-1.42672300
H	6.31808400	-1.51370300	-4.39624700
C	5.25018100	-4.58156500	0.51551000
H	5.19075000	-5.58336500	0.93431300
C	5.58311200	-4.43899600	-0.86613100
C	4.07658800	2.36748000	4.01127600
C	4.15022000	3.67736100	3.47779400
C	4.48289900	3.85058500	2.09261300
C	4.56290600	5.16740600	1.53210400
C	3.90676600	4.82840600	4.26640100
C	3.98861400	6.09919100	3.71140400
H	3.79875400	6.96991900	4.33282200
C	4.31153700	6.27467800	2.36100900
C	5.14278900	4.21897400	-0.65772400
C	5.71974500	3.27153200	-2.84027700
C	6.23831000	-4.09998200	-3.60064800
C	5.83465200	-5.54623400	-1.69504200
C	6.15719900	-5.37075600	-3.04552000
H	6.34725200	-6.24144700	-3.66692100
H	3.82483600	2.24188600	5.06150100
H	3.65546600	4.70193700	5.31599400
H	4.37230000	7.27463100	1.93999900
H	5.97108800	3.39396000	-3.89064400
H	6.48920500	-3.97348400	-4.65033000
H	5.77430400	-6.54618200	-1.27397300
C	5.48036300	4.35420000	-2.05875100
C	4.89560600	5.30994600	0.15040100
H	5.53818300	5.35733000	-2.47333600
H	4.95498700	6.31173000	-0.26845300

BC-BCG-4

C	2.18465100	0.18921200	0.01108900
C	3.49824700	0.81217800	0.53063900
C	3.88571100	2.07333500	0.07273500
C	4.30672700	0.13666700	1.48277900
C	5.04526500	2.67719900	0.53460200
H	3.28481100	2.60239300	-0.65460400
C	5.46164400	0.78980200	1.93447700
C	5.86418600	2.05618200	1.48076500
C	2.39590700	-1.08334800	-0.82944800
C	3.65960600	-1.25683300	-1.40115100
C	1.36979300	-2.02742000	-1.10178200
C	3.94089000	-2.35236700	-2.20054400
H	4.43981300	-0.52841300	-1.22703000
C	1.71246900	-3.14692100	-1.87724300
C	2.98316500	-3.33882900	-2.44517200
C	1.29383500	1.20136400	-0.71824600
C	0.29862400	1.75550900	0.08055400
C	1.38560500	1.60113900	-2.05034100
C	-0.61279700	2.70482900	-0.37011900
C	0.48374900	2.55529700	-2.53033200
H	2.14073500	1.17507100	-2.70308700
C	-0.50267100	3.10510800	-1.70259000
H	-1.38494700	3.10634900	0.27756200
H	0.54653800	2.86869400	-3.56771400
H	-1.19720400	3.83786000	-2.10015000
O	1.39088800	-0.16862100	1.22423000
S	0.37385100	1.03552900	1.69472200
O	1.04514700	1.91216500	2.66115800
O	-0.87744400	0.39743600	2.11133100
O	3.21504500	-4.44558000	-3.19215700
H	4.13840300	-4.43375700	-3.50963100
Br	0.43081600	-4.53081700	-2.21627100

Br	5.68425300	-2.54045200	-2.96928400
C	-0.04554100	-1.87196700	-0.60028900
H	-0.16600600	-2.30058300	0.40082700
H	-0.34647900	-0.82689100	-0.55538600
H	-0.74530800	-2.38393000	-1.26202300
Br	5.53873400	4.40854500	-0.11929300
Br	6.58185800	-0.04611700	3.24097900
O	7.00280200	2.60276100	1.97080100
H	7.14517000	3.47586300	1.55734100
C	3.99291000	-1.23659500	2.02639000
H	4.85984000	-1.89525600	1.91165300
H	3.77307500	-1.18214300	3.09908800
H	3.14534500	-1.70627500	1.53549200
C	-4.54531600	2.72637400	0.39963500
C	-4.46351300	1.71937700	1.37625000
C	-4.42143700	0.34643200	0.98721600
C	-4.46003000	-0.00237600	-0.39910400
C	-4.54370900	1.01315500	-1.38063900
C	-4.58641200	2.37979000	-0.98853200
C	-4.34054500	-0.66935900	1.96866800
C	-4.41400300	-1.37524100	-0.78816400
C	-4.33355700	-2.38222500	0.18852400
C	-4.29813000	-2.03589200	1.57666600
C	-4.28853100	-3.77522300	-0.19687600
C	-4.32389600	-4.09060100	-1.60925600
C	-4.39965000	-3.11901500	-2.55314500
C	-4.45147200	-1.71737400	-2.19566200
C	-4.53474300	-0.71794800	-3.14166000
C	-4.58363500	0.66511200	-2.78097600
H	-4.56335700	-0.97869900	-4.19689900
H	-4.28816300	-5.13807000	-1.89747200
H	-4.42562500	-3.37608200	-3.60894200
C	-4.42088100	2.06185000	2.78346500
C	-4.34178200	1.06213400	3.72948100

C	-4.30113800	-0.32128900	3.36904400
H	-4.31017000	1.32291500	4.78461200
C	-4.22526500	-1.33615900	4.32062000
C	-4.18456700	-2.70314600	3.95274600
C	-4.22033700	-3.05398700	2.56178300
H	-4.19632500	-1.07585400	5.37583700
C	-4.21416800	-4.75142300	0.77565300
H	-4.18100600	-5.79823400	0.48309500
C	-4.17862200	-4.43117900	2.16714800
C	-4.66601900	1.67978300	-3.73232300
C	-4.70927400	3.04680000	-3.36456600
C	-4.66822200	3.39796300	-1.97354600
C	-4.70801600	4.77558600	-1.57883700
C	-4.78998000	4.08518600	-4.32457300
C	-4.82811500	5.41625200	-3.92896400
H	-4.88967800	6.19872100	-4.68019000
C	-4.78753700	5.76524800	-2.57426900
C	-4.58564700	4.11991000	0.78504300
C	-4.46343500	3.46375500	3.14071400
C	-4.10993900	-3.74155000	4.91311200
C	-4.10461500	-5.42104200	3.16266000
C	-4.07124200	-5.07260700	4.51763900
H	-4.01428000	-5.85524200	5.26903400
H	-4.69648500	1.41938700	-4.78749000
H	-4.82106700	3.82427300	-5.37893400
H	-4.81769000	6.81045000	-2.27873900
H	-4.43071900	3.72088000	4.19630200
H	-4.08339500	-3.48065300	5.96760000
H	-4.07337300	-6.46619500	2.86703300
C	-4.54018500	4.43548700	2.19725100
C	-4.66424400	5.09607100	-0.18746100
H	-4.57036900	5.48310700	2.48547100
H	-4.69435100	6.14297200	0.10506200

BC-BCG-5

C	3.94105300	-1.13252600	0.80193700
C	2.56563600	-1.65598900	0.33527800
C	2.46647800	-2.94907400	-0.18328000
C	1.39579300	-0.85874600	0.44852000
C	1.24391600	-3.46842900	-0.58068300
H	3.34556800	-3.57080500	-0.28493700
C	0.17825400	-1.42516500	0.04616500
C	0.06556000	-2.72662200	-0.46887900
C	4.46253100	0.05882200	-0.02164800
C	3.91466600	0.23415000	-1.29596300
C	5.49438600	0.92872700	0.42105800
C	4.33168500	1.26640800	-2.11991200
H	3.15138300	-0.44172300	-1.65725200
C	5.85492800	1.98724300	-0.42818500
C	5.29873900	2.18374500	-1.70273500
C	4.98939500	-2.24418200	0.91677300
C	5.13957300	-2.74788500	2.20486000
C	5.76809600	-2.77929300	-0.10775200
C	6.01618200	-3.77653200	2.53399600
C	6.65781300	-3.81452700	0.19367100
H	5.68527900	-2.39758100	-1.12028000
C	6.78005000	-4.31296300	1.49650000
H	6.11321300	-4.13676200	3.55291400
H	7.26953600	-4.23458800	-0.59860700
H	7.48166000	-5.11359000	1.70678100
O	3.75081200	-0.67990200	2.21420700
S	4.08806100	-1.85618600	3.31238800
O	2.86941200	-2.60409400	3.64156200
O	4.81029900	-1.21526000	4.41464100
O	5.71979200	3.22817100	-2.45594800
H	5.25207900	3.21462500	-3.31305000
Br	7.16490500	3.27499800	0.11772100

Br	3.55408800	1.47017600	-3.85791800
C	6.20467900	0.76410300	1.74268500
H	5.69238800	1.31038900	2.54273000
H	6.27582000	-0.27980200	2.04064700
H	7.22148400	1.15443700	1.67888500
Br	1.15402800	-5.24511600	-1.29250200
Br	-1.44134200	-0.41424800	0.17685500
O	-1.15552100	-3.19034400	-0.82915500
H	-1.06996600	-4.11128100	-1.14140100
C	1.39244600	0.55820800	0.97119600
H	0.93444000	1.23020000	0.23776500
H	0.78962200	0.62523000	1.88423200
H	2.38622000	0.93263400	1.19864800
C	-4.09047700	2.93488600	1.80314400
C	-4.67537800	1.66083100	1.89739400
C	-4.82659900	0.85417300	0.72905000
C	-4.38507600	1.34734600	-0.53914700
C	-3.79252100	2.62918600	-0.63049000
C	-3.64442200	3.42913300	0.53628400
C	-5.41645900	-0.42876300	0.82074100
C	-4.53760800	0.54123200	-1.70787400
C	-5.12381400	-0.73192700	-1.61375300
C	-5.56736100	-1.22729200	-0.34654700
C	-5.28290800	-1.55389300	-2.79270500
C	-4.82393200	-1.02270100	-4.05852800
C	-4.25875800	0.20708900	-4.15033000
C	-4.08512700	1.04977900	-2.98657200
C	-3.51065100	2.30081500	-3.06180900
C	-3.34302300	3.12884800	-1.90781200
H	-3.17182800	2.67924600	-4.02324700
H	-4.94547600	-1.64186400	-4.94358600
H	-3.92011100	0.58895700	-5.10998600
C	-5.12863100	1.15259800	3.17615300
C	-5.69984000	-0.09991700	3.25188700

C	-5.86553500	-0.92867000	2.09807400
H	-6.03855200	-0.47842100	4.21331500
C	-6.44255700	-2.19496000	2.16824300
C	-6.60210400	-3.00727800	1.01911300
C	-6.15828800	-2.51390800	-0.25323500
H	-6.78087900	-2.57401800	3.12947700
C	-5.85962600	-2.80276000	-2.68225700
H	-5.97846000	-3.41914400	-3.57021000
C	-6.31006400	-3.31936900	-1.42917600
C	-2.76507000	4.39468200	-1.97777000
C	-2.60686800	5.20772100	-0.82894500
C	-3.05350100	4.71577500	0.44295100
C	-2.90509400	5.52270900	1.61828000
C	-2.02261700	6.49646500	-0.89090700
C	-1.88435900	7.26863300	0.25557700
H	-1.43435600	8.25516100	0.18742600
C	-2.31766200	6.79436200	1.49890700
C	-3.93466100	3.75834200	2.98157000
C	-4.95865600	1.99711900	4.33915200
C	-7.18599000	-4.29621200	1.08109000
C	-6.89729300	-4.59112500	-1.30983400
C	-7.32706300	-5.06709700	-0.06591900
H	-7.77671200	-6.05379000	0.00223200
H	-2.42569100	4.77322800	-2.93884300
H	-1.68323400	6.87383200	-1.85175600
H	-2.20333100	7.40948500	2.38735200
H	-5.29811700	1.61569500	5.29867500
H	-7.52292900	-4.67471100	2.04234900
H	-7.01403900	-5.20513000	-2.19875800
C	-4.39556100	3.22782600	4.24698200
C	-3.35870800	5.00751100	2.87082700
H	-4.27673900	3.84811800	5.13159500
H	-3.24232800	5.62501700	3.75830300

BC-BCG-6

C	2.49720900	0.40224100	0.58617800
C	3.31052300	-0.87718300	0.29394300
C	3.62058700	-1.74600600	1.34250100
C	3.72094600	-1.20184300	-1.02615900
C	4.31663300	-2.92311700	1.11405300
H	3.32183800	-1.51505800	2.35599800
C	4.40570000	-2.41009100	-1.21501300
C	4.72242000	-3.29316200	-0.17033900
C	3.28264000	1.70393900	0.34175800
C	4.67736000	1.61069800	0.35757600
C	2.66816800	2.97427200	0.17771100
C	5.47272600	2.73106800	0.18033600
H	5.15632800	0.65322300	0.51174700
C	3.51114700	4.07474500	-0.04573900
C	4.91350500	3.99035700	-0.04689700
C	1.85198700	0.38926000	1.97617400
C	0.52450400	-0.02631100	1.97881400
C	2.44808500	0.73850700	3.18683900
C	-0.24794700	-0.13439700	3.13090500
C	1.69564000	0.64023100	4.36079800
H	3.47693700	1.08226100	3.21544300
C	0.36464200	0.20577600	4.33775900
H	-1.28438900	-0.45302500	3.09316100
H	2.15244800	0.91347200	5.30686300
H	-0.20123800	0.14389200	5.26157400
O	1.33146200	0.36792000	-0.34590100
S	0.00041200	-0.33499300	0.31798700
O	-0.02466000	-1.76668400	-0.00137000
O	-1.15311100	0.47290400	-0.08694800
O	5.64462900	5.11117700	-0.25960100
H	6.59415800	4.88554700	-0.23141300
Br	2.78227000	5.81595200	-0.37847500

Br	7.37986300	2.55961400	0.22141100
C	1.17348200	3.17793500	0.23422800
H	0.70944600	3.01586500	-0.74488400
H	0.70058800	2.50391800	0.94612700
H	0.93872600	4.19689500	0.54438800
Br	4.72111200	-4.08603700	2.58101900
Br	4.95218400	-2.93298500	-2.97221700
O	5.39414300	-4.43578000	-0.45023400
H	5.52999500	-4.93895900	0.37550000
C	3.46357100	-0.32073100	-2.22508800
H	4.39828200	-0.13430400	-2.76389500
H	2.78388900	-0.81796400	-2.92685200
H	3.02900500	0.63968900	-1.96270300
C	-6.02834800	1.36295800	0.99388100
C	-5.78301800	-0.00672700	1.18852100
C	-5.11091400	-0.76394800	0.18196000
C	-4.68732300	-0.12546900	-1.02546700
C	-4.93552200	1.25385400	-1.21918400
C	-5.60553000	2.00435000	-0.21352300
C	-4.86295700	-2.14334800	0.37555800
C	-4.01514600	-0.88271100	-2.03209000
C	-3.76961500	-2.25233800	-1.83735800
C	-4.19280600	-2.89382000	-0.63007500
C	-3.08930500	-3.02636900	-2.85188800
C	-2.67513400	-2.34686400	-4.06096800
C	-2.91147700	-1.02448900	-4.24967000
C	-3.58996700	-0.22578500	-3.25145700
C	-3.83795800	1.11892600	-3.42732000
C	-4.50907500	1.90198600	-2.43642800
H	-3.51718200	1.60852700	-4.34375700
H	-2.16403200	-2.93009700	-4.82260300
H	-2.59283100	-0.53172800	-5.16466900
C	-6.20799000	-0.66358800	2.40799100
C	-5.96078900	-2.00853600	2.58355200

C	-5.28991500	-2.79168500	1.59252800
H	-6.28169600	-2.49814000	3.49995100
C	-5.03783700	-4.15135500	1.76352800
C	-4.37243200	-4.91761300	0.77558200
C	-3.94539900	-4.27735900	-0.43561200
H	-5.35916800	-4.64158800	2.67931600
C	-2.85720300	-4.37078400	-2.64463000
H	-2.34536100	-4.95035300	-3.40926600
C	-3.27024300	-5.03378100	-1.44889000
C	-4.76211800	3.26139400	-2.60789800
C	-5.42754800	4.02769400	-1.61999700
C	-5.85377200	3.38770500	-0.40837500
C	-6.52883300	4.14423600	0.60490900
C	-5.68856500	5.41006500	-1.78524600
C	-6.34421900	6.13188300	-0.79590500
H	-6.53568000	7.19140500	-0.94156100
C	-6.76265100	5.51340800	0.38777700
C	-6.70815400	2.13713500	2.00863100
C	-6.88587800	0.13528400	3.40650600
C	-4.11232200	-6.30021100	0.94037100
C	-3.03733500	-6.40315500	-1.23221700
C	-3.45661300	-7.02192900	-0.04898900
H	-3.26566000	-8.08159100	0.09630600
H	-4.44129400	3.75147000	-3.52394300
H	-5.36695200	5.89932300	-2.70070400
H	-7.27437900	6.08976700	1.15381100
H	-7.20415200	-0.35737500	4.32169500
H	-4.43449600	-6.78966000	1.85553100
H	-2.52538600	-6.97941200	-1.99817500
C	-7.12185600	1.45775800	3.21796100
C	-6.94099200	3.48142300	1.80107400
H	-7.63222500	2.04116700	3.97996200
H	-7.45240800	4.06107000	2.56595000

BC-COOH-BCG

C	-3.55440100	1.57660600	0.07771400
C	-2.23342900	2.37097300	-0.00003800
C	-2.14252700	3.47016800	-0.85697300
C	-1.12015100	2.02526200	0.81077700
C	-0.98359400	4.22870200	-0.92576400
H	-2.97805400	3.74959800	-1.48434500
C	0.03159900	2.81814400	0.71161400
C	0.13345800	3.92705000	-0.14333100
C	-3.41722500	0.10753000	-0.35921900
C	-2.35502600	-0.20010000	-1.21366100
C	-4.33681300	-0.91249300	0.00132800
C	-2.14861800	-1.49055400	-1.67791500
H	-1.67351600	0.57700500	-1.53322100
C	-4.05554300	-2.21539900	-0.43855200
C	-2.96841000	-2.54893300	-1.27074700
C	-4.71602300	2.26965600	-0.64238200
C	-5.54273500	3.00918300	0.19707400
C	-5.00943100	2.22617600	-2.00436200
C	-6.64913200	3.72688300	-0.24601100
C	-6.11380300	2.93975300	-2.47848800
H	-4.39291500	1.64478100	-2.68229700
C	-6.92505000	3.68416300	-1.61316100
H	-7.27917400	4.28245200	0.44078500
H	-6.35015300	2.90807900	-3.53751100
H	-7.78202400	4.22345600	-2.00356200
O	-3.96060500	1.61003500	1.51724800
S	-4.97707100	2.85302500	1.86535100
O	-4.20935800	4.02540800	2.30086000
O	-5.99353800	2.33012100	2.78244800
O	-2.80451900	-3.83388700	-1.64305800
H	-1.90686200	-4.02419600	-2.00831100
Br	-5.17348100	-3.67945300	0.09742400

Br	-0.71250400	-1.81656300	-2.89529700
C	-5.57582600	-0.64801000	0.82340200
H	-5.36505500	-0.69401700	1.89754500
H	-6.00066800	0.33108600	0.60734900
H	-6.34293900	-1.39223100	0.60646900
Br	-0.90155700	5.73432600	-2.10836000
Br	1.56894200	2.39665000	1.76861000
O	1.28669000	4.63888100	-0.16831700
H	1.19517300	5.37162600	-0.80724600
C	-1.10780600	0.85705200	1.76792200
H	-0.28502400	0.17638800	1.52453500
H	-0.93555600	1.20876000	2.79152000
H	-2.03246500	0.28747200	1.76024700
C	2.02309200	-1.99720200	2.56547300
C	3.01533200	-1.00841100	2.44650100
C	3.67595000	-0.80632100	1.19901500
C	3.33337200	-1.61687700	0.07170800
C	2.33476200	-2.61579800	0.19153200
C	1.67048500	-2.80567700	1.43685600
C	4.66997900	0.19406500	1.07640500
C	3.99826400	-1.40475300	-1.17259000
C	4.98537800	-0.41197100	-1.29360500
C	5.32941700	0.39629500	-0.16651100
C	5.65591300	-0.19542900	-2.55553900
C	5.27869200	-1.02519200	-3.67850100
C	4.32355100	-1.98146600	-3.56248200
C	3.64015300	-2.21857600	-2.30960700
C	2.66620200	-3.18641600	-2.18305900
C	1.98882700	-3.43416200	-0.94712300
H	2.41782900	-3.77646100	-3.05928600
H	5.78406500	-0.86037500	-4.62646300
H	4.04964700	-2.59621000	-4.41578200
C	3.36915800	-0.18261800	3.58362200
C	4.33561600	0.79165100	3.44820800

C	5.01632300	1.01419100	2.21154400
H	4.59809500	1.40970200	4.30329700
C	5.99859500	1.99290100	2.07204500
C	6.66691400	2.20876500	0.84319500
C	6.32464000	1.39879000	-0.28890200
H	6.25990700	2.61018100	2.92795100
C	6.62294200	0.78492200	-2.65527700
H	7.12693300	0.94686600	-3.60506500
C	6.98555400	1.60250000	-1.54288800
C	0.97548100	-4.40140400	-0.80925800
C	0.28042500	-4.59210900	0.42740100
C	0.65292500	-3.79015700	1.55808400
C	-0.00622600	-3.97852100	2.81914100
C	-0.73532700	-5.56486500	0.60130700
C	-1.36250300	-5.73405200	1.82943500
H	-2.13827200	-6.48781800	1.93002400
C	-1.01013400	-4.95467500	2.93157600
C	1.34951000	-2.20120700	3.82831900
C	2.68182700	-0.41412000	4.83479700
C	7.66513300	3.20181500	0.69178500
C	7.96897200	2.60281100	-1.63801300
C	8.30051500	3.38955500	-0.52927700
H	9.06361300	4.15635300	-0.62670400
H	-1.03225200	-6.18919800	-0.23307200
H	-1.50717000	-5.09570000	3.88725100
H	2.95268700	0.20459300	5.68622800
H	7.92719600	3.81718800	1.54811300
H	8.47228600	2.76020900	-2.58805100
C	1.72800300	-1.37029700	4.95062100
C	0.37286800	-3.16798100	3.92975800
H	1.21908700	-1.53305800	5.89708400
H	-0.13384600	-3.32276500	4.87922500
C	0.55801700	-5.21631400	-1.98807100
O	1.47605400	-6.02313300	-2.55878300

H	2.30749100	-6.01786400	-2.04746000
O	-0.56755100	-5.20854300	-2.47278900

BC-OH-BCG

C	-3.29146600	1.46921700	0.56812900
C	-2.01396000	2.30781700	0.35317500
C	-2.11991100	3.60553400	-0.15182100
C	-0.73213500	1.78722200	0.67430900
C	-0.99263900	4.38788300	-0.35307200
H	-3.08622400	4.02413000	-0.39817900
C	0.38194700	2.60646500	0.44523700
C	0.28863300	3.90997300	-0.06812300
C	-3.31898800	0.17869800	-0.27281900
C	-2.53376000	0.16834800	-1.42957400
C	-4.12265600	-0.94971200	0.03611700
C	-2.47188000	-0.95016300	-2.24511400
H	-1.96302400	1.04400800	-1.70768600
C	-3.97741100	-2.08427400	-0.78065400
C	-3.15001900	-2.12700500	-1.91604800
C	-4.58206400	2.27776500	0.40196400
C	-5.13899000	2.70972400	1.60121600
C	-5.22482900	2.60238100	-0.79158600
C	-6.30668500	3.46197800	1.68295300
C	-6.39751700	3.36092500	-0.74020500
H	-4.82259300	2.26764200	-1.74230700
C	-6.93488000	3.78800800	0.48044300
H	-6.71874500	3.77107000	2.63801000
H	-6.90510000	3.61548300	-1.66541600
H	-7.85145600	4.36871200	0.49524800
O	-3.29276300	1.08994300	2.01458900
S	-4.14644700	2.13902500	2.94872200
O	-3.26036900	3.18987900	3.46225600
O	-4.88671100	1.33753900	3.92671900
O	-3.07774600	-3.26090000	-2.65617500
H	-2.16719600	-3.37496500	-3.00910800
Br	-4.91755100	-3.70259900	-0.37009900

Br	-1.44563700	-0.87490900	-3.85456100
C	-5.10056700	-0.97532200	1.18623400
H	-4.62373400	-1.30355500	2.11633000
H	-5.54157600	0.00464100	1.36153000
H	-5.91684600	-1.66740700	0.97430100
Br	-1.18084900	6.16795300	-1.03514400
Br	2.13861400	1.94602200	0.81738900
O	1.42130900	4.62878500	-0.26062300
H	1.18723700	5.49970600	-0.63506400
C	-0.50374100	0.40535800	1.23777900
H	0.14125400	-0.17709400	0.57136300
H	0.01481800	0.47251000	2.20075900
H	-1.42257100	-0.15239500	1.39264300
C	2.11043500	-2.97828600	2.06797500
C	3.20488100	-2.09965100	2.13164800
C	3.69083900	-1.47906600	0.94273100
C	3.06564900	-1.75561700	-0.31453700
C	1.96838000	-2.65008200	-0.37864600
C	1.48310300	-3.25686200	0.81320000
C	4.78625700	-0.58402300	1.00627900
C	3.55332300	-1.12488400	-1.49876100
C	4.63756600	-0.23368800	-1.43355300
C	5.26501600	0.04251700	-0.17811900
C	5.12973500	0.41152700	-2.63001500
C	4.46754300	0.11575900	-3.88223700
C	3.41573400	-0.73851900	-3.94469700
C	2.90805200	-1.40042600	-2.76303000
C	1.84019500	-2.27104000	-2.81563000
C	1.33786400	-2.92560000	-1.64909500
H	1.36379100	-2.42572000	-3.77828600
H	4.83696300	0.60258200	-4.78109700
H	2.92902300	-0.94727400	-4.89400800
C	3.84618300	-1.81427200	3.39859800
C	4.91666500	-0.94717000	3.44529100

C	5.42214500	-0.30477200	2.27227800
H	5.39540300	-0.73595900	4.39851900
C	6.50011100	0.57726600	2.31399100
C	6.99047400	1.21356300	1.14795800
C	6.36199400	0.94079600	-0.11326600
H	6.97897700	0.78727000	3.26731900
C	6.19690600	1.28296400	-2.54784100
H	6.56333000	1.76822700	-3.44939800
C	6.84128500	1.57418800	-1.30705300
C	0.26056000	-3.82083400	-1.67993700
C	-0.25538100	-4.43006800	-0.50088300
C	0.37414400	-4.13927600	0.75276900
C	-0.11993700	-4.74843700	1.95280000
C	-1.35571000	-5.31798000	-0.52840400
C	-1.82689000	-5.89303800	0.64571100
H	-2.67746300	-6.56701900	0.60606300
C	-1.22063800	-5.61932800	1.87354200
C	1.60481400	-3.60264800	3.27022700
C	3.32445800	-2.45983800	4.58397500
C	8.08207900	2.11578500	1.18126700
C	7.92725400	2.46272500	-1.21547200
C	8.53422300	2.72534300	0.01782500
H	9.37207400	3.41567600	0.06397100
H	-1.83822800	-5.54068100	-1.47150500
H	-1.59513000	-6.07996200	2.78369600
H	3.80731100	-2.24630200	5.53412100
H	8.56062100	2.32572300	2.13407500
H	8.29175200	2.94672700	-2.11755000
C	2.26396400	-3.30299500	4.52340400
C	0.52360400	-4.45548900	3.19256000
H	1.88284200	-3.77714200	5.42413900
H	0.14346800	-4.92481200	4.09675000
O	-0.38983700	-4.14261900	-2.85148500
H	0.20275800	-4.03181700	-3.61484000
